# 电视胸腔镜联合前/后径路在肺上沟瘤切除术中的临床应用

**DOI:** 10.3779/j.issn.1009-3419.2015.11.07

**Published:** 2015-11-20

**Authors:** 嘉 焦, 浩 黄, 雷 田, 庆琛 吴, 明建 葛

**Affiliations:** 400016 重庆，重庆医科大学附属第一医院胸外科 Department of Thoracic Surgery, the First Affiliated Hospital, Chongqing Medical University, Chongqing 400016, China

**Keywords:** 肺上沟瘤, 胸腔镜, 微创手术, Superior sulcus tumors, Video-assisted thoracic surgery, Minimally invasive surgery

## Abstract

**背景与目的:**

肺上沟瘤的外科手术治疗具有挑战性。胸腔镜辅助下手术治疗肺上沟瘤的临床研究较少。本研究旨在初步评估胸腔镜应用于肺上沟瘤切除术中的可行性及安全性。

**方法:**

2010年1月-2013年6月在重庆医科大学附属第一医院胸心外科确诊的肺上沟瘤患者10例，最终有6例接受外科手术治疗，包括胸腔镜联合前径路3例及联合后径路3例。研究指标包括：①围手术期死亡率；②手术对肿瘤切除的完整性；③手术的一般资料及术后并发症；④手术后1年复发及转移情况。

**结果:**

无围手术期死亡患者。肿瘤及受累胸壁均为完整切除。手术平均时间为242 min。术中平均出血量为308 mL。平均住院时间为14 d。1例患者术后出现肺部感染，经使用抗生素治愈。术后无严重并发症。所有患者随访1年均无局部复发或远处转移。

**结论:**

胸腔镜联合前/后径路行肺上沟瘤切除术具有实用价值，符合术者视野角度和操作习惯，便于确定胸壁切除的准确范围，有利于肺上沟瘤手术的安全、规范实施。

## Abstract (en)

**Background and objective:**

The surgical resection for pancoast tumors remains challenging. There are only few reports explaining the use of VATS in the treatment of Pancoast tumors. The aim of this study is to assess whether the use of video-assisted thoracoscopic surgery (VATS) for the surgical treatment of Pancoast tumors was feasible and safe.

**Methods:**

Between Janunary 2010 and June 2013, ten patients who were diagnosed as Pancoast tumors were recruited. Six patients were accepted for surgical treatment either through an anterior (*n*=3) or a posterior approach (*n*=3) combined with VATS. The observation index of this study included: Operation safety and mortality; The integrity of the tumor resection; General data of operation and postoperative complications; Tumor recurrence and metastasis at twelve months after operation.

**Results:**

There were no perioperative deaths. The average time of operation time was 242 min. The radical en bloc resection of the involved chest wall were done in each patients. The average amount of blood loss was 308 mL and the average time of hospital stay was 14 d. Only one patient had postoperative pneumonia and recovered after use of antibiotics. There was none of severe postoperative complications. No patient developed a local recurrence or distant metastasis within twelve months.

**Conclusions:**

The use of VATS has practical value in the management of Pancoast tumors. It is useful to make an accurate extent of the resection of chest-wall and provides a better exposure. Anterior or posterior approach with VATS surgery can facilitate the safety management of Pancoast tumors.

肺上沟瘤，也称为Pancoast瘤，是指原发于肺尖部的肿瘤，由于位于胸腔顶部，常常侵犯壁层胸膜、胸内筋膜的淋巴及第1-3肋骨、椎体、臂丛神经、锁骨下血管、星状神经节、交感神经链，因而产生剧烈的胸痛及肩部、手部疼痛和Horner综合征^[1]^。在1950年之前，肺上沟瘤被认为无法手术，或仅为减轻疼痛而做姑息性切除再加放射治疗，因此放射治疗曾被认为是治疗肺上沟瘤的常规治疗手段。直至1956年，Chardack和Maclanm^[2]^首先报道包括肺叶及胸壁切除以后辅助以放射治疗而获得5年生存的病例。

近年来，采用术前放化疗结合手术切除的联合治疗方案获得了较高的手术切除率和病理缓解率，使得肺上沟瘤患者的局部控制及术后总生存率均得以提高^[3]^。根据肿瘤病变累及部位不同，高位后外侧切口(Shaw-Paulson途径、后径路)^[4]^、经锁骨-胸途径^[5]^及经胸骨柄的L形切口(Grunenwald-Spaggiari途径、前径路)^[6]^均为切除肺上沟瘤的经典手术路径。传统的高后外侧切口虽然有优点，但对胸廓入口处的某些结构(如臂丛神经及锁骨下动静脉)显露欠佳。前入路能够在颈-胸交界区域整块游离及切除病变过程中提供良好的手术视野，有时需对多根即使未受累的肋骨进行切除以便获得满意的显露，但该切口对肺门及纵隔的显露欠佳，需要增加另一切口来完成肺叶切除及淋巴结清扫^[3]^。目前，国外有通过利用胸腔镜辅助(video-assisted thoracoscopic surgery, VATS)下手术治疗肺上沟瘤的个案报道^[7-10]^，这里我们报道一组电视胸腔镜联合前径路或后径路肺上沟瘤切除的病例。

## 1 材料和方法

### 1.1 一般资料

2010年1月-2013年6月间于重庆医科大学附属第一医院胸心外科确诊的10例肺上沟瘤患者被纳入此临床研究，所有患者均接受新辅助化疗(化疗方案，TP方案：紫杉醇：140 mg/m^2^，奈达铂：75 mg/m^2^，2个周期)，有2例患者不能耐受化疗反应而被排除，1例患者拒绝接受手术治疗，另有1例患者因发现远处转移而被排除。最终有6例患者接受了手术治疗，本临床研究通过了医院伦理委员会的审查，所有患者入院均签署了知情同意书。

### 1.2 观察指标

①手术的安全性及围手术期死亡率；②手术对肿瘤切除的完整性；③手术的一般资料包括手术时间、术中失血及输血情况、术后并发症(如术后出血、肺部感染、切口感染及肺栓塞等)；④手术后1年复发及转移情况。

### 1.3 手术方法

#### 1.3.1 前径路

如果肺上沟瘤主要位于胸廓入口的前部，与锁骨下血管等关系较密切时，选择Grunenwald-Spaggiari途径：①取侧卧位，于腋前线第7肋间及腋后线第7、9肋间分别取1.5 cm切口作为观察孔及辅助操作孔(图 1)，首先在胸腔镜下探查病变大小、受累情况及确定肋间，常规在胸腔镜下完成肺门解剖分离及肺叶切除，对病肺相对正常的肺组织行机械切割以后从腔镜操作切口(第4肋间腋前线)取出，在VATS下完成各站淋巴结的清扫，关闭各个操作孔；②取平卧位，沿患侧胸锁乳突肌前缘切口，自胸骨上窝至胸骨柄沿胸骨中线部分劈开，根据胸腔镜下探查肋骨受累范围确定目的肋间，将切口向外侧延长，完成L形切口制作，锁骨下静脉被部分切除以后无需重建，锁骨下动脉被部分切除以后可行断端直接吻合或以6 mm-8 mm的人工血管重建，完成受累胸壁组织游离及切除后，将瘤体从L形切口取出，以钢丝缝合胸骨，安放胸引管及胸壁引流，关闭切口。

**1:**
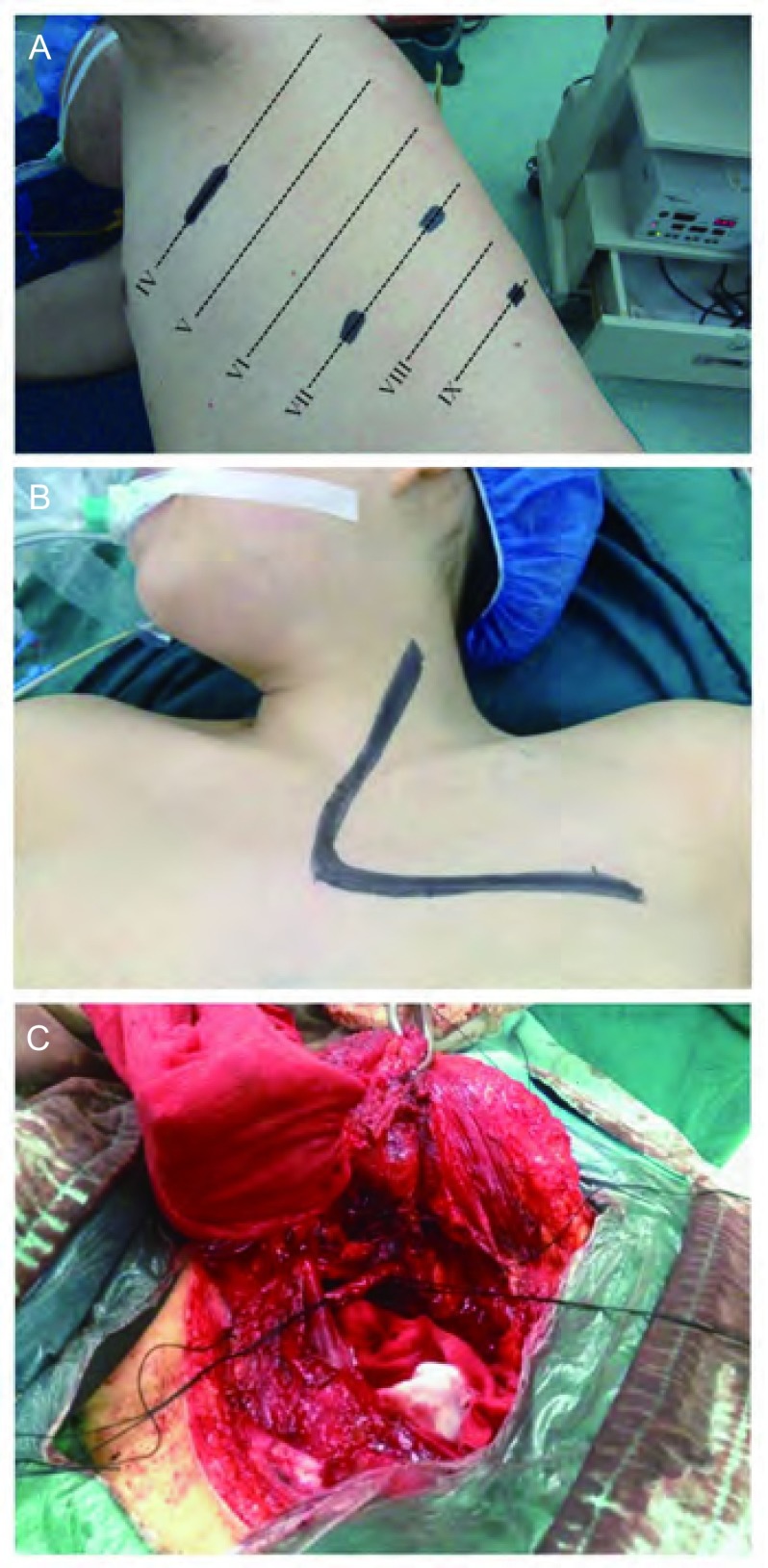
病例2图片。A：胸腔镜肺叶切除及淋巴结清扫的皮肤切口设计；B：前径路的L型切口；C：病变切除后的术野展示。 Pictures of case 2. A: The design of incisions of video-assisted thoracoscopic surgery (VATS) lobectomy and systematic lymph nodes dissection; B: L-shaped incision in anterior approach; C: View after surgical resection.

#### 1.3.2 后径路

如果上沟瘤主要位于胸廓入口的后部，取高后外侧切口与胸腔镜相结合办法实施：①取侧卧位，于腋前线第7肋间及腋后线第7、9肋间分别取1.5 cm切口作为观察孔及辅助操作孔(图 2)，首先在胸腔镜下探查病变大小、受累情况及确定肋间，常规在胸腔镜下完成肺门解剖分离及肺叶切除；②于棘突与肩胛骨后沿之间制作高后外侧切口，后端延及颈根部，前端绕肩胛角，根据胸腔镜下观察的结果，在受累肋骨下方正常一肋骨的肋间隙切开，于受累肋骨距肿瘤边缘至少2 cm-3 cm切断，受累胸壁游离以后，于起始部结扎并切断受累的神经根；切断受累神经根的臂丛神经端；将游离的胸壁向脚侧牵引以便从锁骨下动脉分离肺叶及壁层胸膜；③待胸廓入口区的分离完成后，改在VATS下进行肺门的分离，切除病肺，将其从后外侧切口取出，然后在满意的显露下通过VATS完成肺门及纵隔各站淋巴结清扫术，如胸壁缺损完全位于肩胛骨后方，则无需行胸壁重建；若第4肋以下的肋骨被切除，则需运用不可吸收的补片来重建胸壁；安放引流管及关闭胸部切口。

**2:**
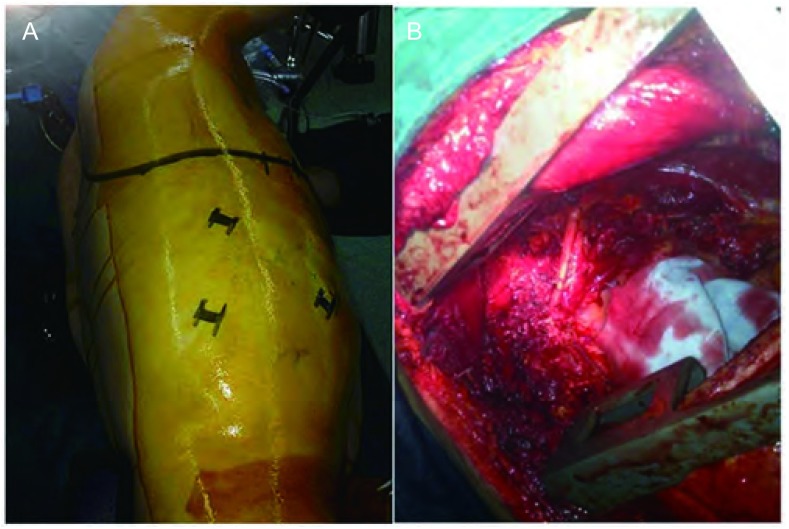
病例6图片。A：胸腔镜联合后径路的皮肤切口设计；B：病变切除后的术野展示。 Pictures of case 6. A: The design of incisions in posterior approach with VATS; B: View after surgical resection.

## 2 结果

本组采用联合径路行手术切除肺上沟瘤共6例，采用VATS联合改良后径路(Shaw-Paulson approach)及VATS联合经胸骨柄前入路(Grunenwald approach)各3例。手术在化疗结束后3周进行。所有手术均为上肺叶及受累胸壁完整切除。无围手术期死亡病例。1例行锁骨下动脉重建(病例1)，2例行T1神经根节段性切除(病例3、4)，2例行锁骨下静脉节段性切除(病例5、6)，无行胸壁重建的病例。手术平均时间为242 min，术中平均出血量为308 mL。平均住院时间为14 d。有1例患者术后出现肺部感染，经使用抗生素治愈。术后病理分期：Ⅱb期3例，Ⅲa期2例，Ⅲb期1例。随访1年内无局部复发及远处转移病例(表 1)。

**1:** 6例肺上沟瘤围手术期的特点 Perioperative characteristics of the six patients with Pancoast tumors

Case No.	1	2	3	4	5	6
Age (yr)	62	53	55	49	57	62
Gender	M	F	F	M	M	M
Comorbidities	Hypertension	Diabetes	Hypertension	COPD	/	/
Approach	Posterior	Anterior	Posterior	Anterior	Anterior	Posterior
Operation	Ⅰ, Ⅱ, Ⅲ rib	Ⅰ, Ⅱ rib	Ⅰ, Ⅱ, Ⅲ rib	Ⅰ, Ⅱ, Ⅲ rib	Ⅰ, Ⅱ, Ⅲ rib	Ⅰ, Ⅱ, Ⅲ rib
	resection LUL Subclavian artery Reconstruction Lymphadenectomy	resection LUL Lymphadenectomy	resection RUL T1 Nerve root Stage resection Lymphadenectomy	resection RUL T1 Nerve root Stage resection Lymphadenectomy	resection RUL Subclavian vein Stage resection Lymphadenectomy	resection RUL Lymphadenectomy
Operative time (min)	275	220	235	240	265	215
Blood loss (mL)	220	200	500	380	250	300
Hospital stay (d)	13	10	25	11	14	12
Complications	No	No	pneumonia	No	No	No
Histological type Pathological stages	Adenocarcinoma T4N2M0 Ⅲb	Adenocarcinoma T3N0M0 Ⅱb	Adenocarcinoma T4N1M0 Ⅲa	Squamouscarcinoma T4N0M0 Ⅲa	Adenocarcinoma T3N0M0 Ⅱb	Adenocarcinoma T3N0M0 Ⅱb
Follow-up (12 months)	Alive	Alive	Alive	Alive	Alive	Alive
M: male; F: female; COPD: chronic obstructive pulmonary diseases; LUL: left upper lobectomy; RUL: right upper lobectomy.						

## 3 讨论

肺上沟瘤的外科手术具有挑战性。术前诱导化疗/放疗结合外科根治性切除被认为是本病的标准治疗方案^[3]^。外科手术时除了上肺叶切除及淋巴结清扫以外，还需对受累胸壁行整块根治性切除(radical *en*-*bloc* resection)。国内外学者针对肺上沟瘤的部位设计了不同的手术入路。此类手术的主要难点在于术野的显露^[1]^。

随着微创胸部外科的发展，电视胸腔镜被广泛应用于早期非小细胞肺癌的手术治疗之中，并被证实具有手术创伤小、术后恢复快等优点^[11-12]^。迄今为止，很少有将电视胸腔镜运用于肺上沟瘤手术过程的报道，有些学者将其运用于实施切除术之前的胸腔探查，以决定是否进一步手术，或者参考选择合适的手术路径^[13]^。Nakajima等^[7]^曾报道过在1例肺上沟瘤患者术中利用胸腔镜进行胸腔粘连的松解。Truin等^[8]^及Linden等^[9]^各有1例前径路切除Pancoast瘤术中采用c-VATS途径行肺叶切除术的报道。在Caronia等^[10]^的研究中，他们报道了一组来自4个不同肿瘤中心的7例肺上沟瘤病例，所有患者均在胸腔镜下联合前/后入路完成肺叶及受累胸壁的完整切除、淋巴结清扫。他们的初步经验显示应用胸腔镜辅助肺上沟瘤是安全、可靠的。在国内尚未见有此类手术的相关报道。我们采用电视胸腔镜下探查胸腔、选择合适的手术路径，并通过胸腔镜下直视探查来决定肿瘤及受累胸壁的切除范围。肺叶切除及淋巴结清扫均在胸腔镜下完成，并同时与前/后径路相结合的方式完成胸壁受累结构切除的手术治疗。在本研究中，所有患者均为肺叶及受累胸壁的完整切除，围手术期未出现严重并发症及死亡病例。随访1年中，所有患者均未见局部复发及远处转移。我们充分利用VATS的优势，保证了手术的安全与规范。

对于肺上沟瘤手术，在决定切除范围及选择具体的手术途径之前先在胸腔镜下探查病变的相关情况十分有益，比如肿瘤有否胸膜播散、病变在胸廓入口的准确部位、受累肋骨范围、纵隔淋巴结是否肿大等，以便决定下一步的手术方案(如肺叶切除或局部姑息性切除)。虽然Martinod等^[14]^认为不同手术途径并不影响肺上沟瘤的术后5年生存率。但是我们认为不同的手术径路也应该运用于不同情形的肺上沟瘤，而针对每例患者术中先在胸腔镜下探查，则有助于选择最佳的手术径路。对于存在胸膜粘连的病例，VATS更是显示出独特的优势。

在前径路中，肺门的分离及各站淋巴结的清扫均在胸腔镜下完成，而标本的取出则可通过L形切口取出，由于肺上沟瘤属于周围型肺癌，一般情况下肺门结构的分离及切断在VATS下完成较为容易及安全。为了便于上纵隔淋巴结的清扫，我们习惯于在胸腔镜下先对病肺相对正常的肺组织行机械切割以后从腔镜操作切口取出，然后于VATS下完成各站淋巴结的清扫，关闭所有操作孔以后，将患者由侧卧位改为平卧位，待颈-胸交界区域整块切除完成以后才将瘤体连同受累胸廓结构(如肋骨等)从L形切口取出。如仅仅通过L型单一切口完成全部手术，则需要在较高的肋间(例如第2肋间)行肺叶切除术，对肺门和纵隔区域的显露较困难，不符合通常的视野角度及操作习惯。若联合后外侧切口完成肺叶切除，要从更低水平切开肋间隙从而造成更大的创伤。我们采用VATS途径来实施肺叶切除及淋巴结清扫术。相比于L型单一切口手术，通过胸腔镜途径行肺叶切除可在术中提供正常解剖视角，操作较为简便，同时胸壁的微创又能避免传统高位后外侧切口所带来的巨大创伤，减轻患者术后的疼痛。

如果肺上沟瘤主要位于胸廓入口后方，传统的方法是采用向后上方延长的高后外侧切口，通常需经第4或5肋间进入胸腔探查肋骨受累的范围，为了便于行肺叶切除术，这种方法需切除较多的正常肋骨，有时需行胸壁缺损的重建，创伤较大。我们选择VATS与后径路相结合的方法对这类肺上沟瘤进行治疗，先在胸腔镜下探查肋骨等受累范围，仅在受累肋骨下方的下一肋骨的肋间隙切开，切除肋骨范围较小，胸壁缺损亦较小，由于肩胛骨的遮挡，胸壁缺损往往无需重建。由于肺叶切除及淋巴结清扫均在胸腔镜下完成，胸腔镜联合改良后的高后外侧切口较传统高后外侧切口(约40 cm)相比，大大减小了手术的创伤，有利于患者术后快速恢复。

值得注意的是，我们报道的VATS联合前/后径路行肺上沟瘤切除术时的操作顺序与国外文献报道不同，文献报道VATS与前入路联合时均是先行胸廓入口的分离解剖，然后才完成肺叶切除及淋巴结清扫术。我们认为如首先行胸廓入口解剖，则受累肋骨等胸壁结构被切除以后不利于胸腔镜下肺门显露，更不利于手术标本的取出。而如果首先完成胸腔镜下探查及肺叶切除，一方面可帮助我们判断病变能否完整切除，另一方面可以在胸腔镜直视下确定受累胸壁的切除范围。因此。我们推荐的操作顺序如下：①VATS+前径路：肺叶切除→淋巴结清扫→胸廓入口；②VATS+后径路：肺叶切除→胸廓入口→淋巴结清扫。

我们研究的不足之处在于病例数较少，并且缺乏对照研究，由于此类患者发病率低，并且部分患者不适于手术治疗，造成病例积累困难，如能够联合国内多个肿瘤中心，完成联合胸腔镜对照传统开胸手术治疗肺上沟瘤的前瞻性临床对照研究，则能够进一步明确胸腔镜在肺上沟瘤手术治疗中的应用价值。

我们的初步经验表明，VATS联合前/后径路行肺上沟瘤切除术具有实用价值，可以减少骨性胸廓的切除范围，从而减少传统手术途径的并发症，由于胸腔镜的良好显露效果，符合术者视野角度和操作习惯，便于确定胸壁切除的准确范围，有利于肺上沟瘤手术的安全、规范实施。
